# The administration of dextrose during in-hospital cardiac arrest is associated with increased mortality and neurologic morbidity

**DOI:** 10.1186/s13054-015-0867-z

**Published:** 2015-04-10

**Authors:** Teng J Peng, Lars W Andersen, Brian Z Saindon, Tyler A Giberson, Won Young Kim, Katherine Berg, Victor Novack, Michael W Donnino, for the American Heart Association’s Get With The Guidelines®-Resuscitation Investigators

**Affiliations:** Department of Emergency Medicine, Beth Israel Deaconess Medical Center, One Deaconess Road, W/CC 2, Boston, MA 02215 USA; Department of Anesthesiology, Aarhus University Hospital, Aarhus, Denmark; Department of Medicine, Division of Critical Care, Beth Israel Deaconess Medical Center, Boston, MA USA; Clinical Research Center, Soroka University Medical Centers, Beer-Shave, Israel

## Abstract

**Introduction:**

Dextrose may be used during cardiac arrest resuscitation to prevent or reverse hypoglycemia. However, the incidence of dextrose administration during cardiac arrest and the association of dextrose administration with survival and other outcomes are unknown.

**Methods:**

We used the Get With The Guidelines®-Resuscitation national registry to identify adult patients with an in-hospital cardiac arrest between the years 2000 and 2010. To assess the adjusted effects of dextrose administration on survival, we used multivariable regression models with adjustment for multiple patient, event, and hospital characteristics. We performed additional analyses to examine the effects of dextrose on neurological outcome and return of spontaneous circulation.

**Results:**

Among the 100,029 patients included in our study, 4,189 (4.2%) received dextrose during cardiac arrest resuscitation. The rate of dextrose administration increased during the study period (odds ratio 1.11, 95% confidence interval (CI) 1.09-1.12 per year, *P* <0.001). Patients who received dextrose during resuscitation had lower rates of survival compared with patients who did not receive dextrose (relative risk 0.88, 95% CI 0.80-0.98, *P* = 0.02). Administration of dextrose was associated with worse neurological outcome (relative risk 0.88, 95% CI 0.79-0.99, *P* = 0.03) but an increased chance of return of spontaneous circulation (relative risk 1.07, 95% CI 1.04-1.10, *P* <0.001).

**Conclusions:**

In this dataset, the administration of dextrose during resuscitation in patients with in-hospital cardiac arrest was found to be associated with a significantly decreased chance of survival and a decreased chance of good neurological outcome.

**Electronic supplementary material:**

The online version of this article (doi:10.1186/s13054-015-0867-z) contains supplementary material, which is available to authorized users.

## Introduction

In-hospital cardiac arrest (IHCA) is one of the leading causes of death in the United States, with an incidence of over 200,000 patients per year and a mortality rate of more than 75% [1]. Over the past decade, there have been enhancements to cardiac life support interventions, increased quality-improvement efforts, and improved IHCA survival trends [2]. Nevertheless, the mortality rate for IHCA patients remains extremely high [1,3,4].

In 2005, the American Heart Association guidelines for advanced cardiac life support (ACLS) [5] listed hypoglycemia as a reversible cause of cardiac arrest but removed it upon the publication of the current 2010 ACLS guidelines [6]. Pre-2005 editions of the ACLS guidelines have never included hypoglycemia as a reversible cause of cardiac arrest, and the provision of dextrose during cardiac arrest in the absence of confirmed hypoglycemia is not suggested in the current guidelines [7]. To date, the current 2010 ACLS guidelines recommend the use of dextrose with insulin to treat severe hyperkalemia and suggest that insulin with dextrose can be considered for severe beta-blocker overdose, but neither support nor discourage the use of dextrose for any other condition [6,8].

The use of dextrose in cardiac arrest has not been adequately studied in the clinical setting, and the incidence of dextrose administration remains unknown [9]. Experimental evidence has suggested that dextrose administration might be harmful. Animal studies have shown that administering dextrose before, during, or after cardiac arrest leads to higher rates of mortality and worse neurological outcome [10-12]. In a study using pigs, hyperglycemia prior to cardiac arrest was associated with increased ischemic brain injury and increased markers of cerebral injury [13]. Similarly, human studies have shown that higher post-arrest blood glucose levels are associated with increased mortality and poor neurological outcome [14-20]. Hyperglycemia is also an independent predictor of mortality in myocardial infarction and stroke [21,22].

We hypothesized that the administration of dextrose during cardiac arrest resuscitation would be associated with higher post-arrest mortality and worse neurological outcome. To test this hypothesis, we used a large national registry of IHCAs to establish the rate of dextrose administration during cardiac arrest. We then compared the survival with discharge of patients who received dextrose with patients who did not receive dextrose during cardiac arrest resuscitation. Secondarily, we assessed the association between dextrose administration and return of spontaneous circulation (ROSC) and neurological outcome.

## Methods

### Data source

The Get With The Guidelines®-Resuscitation (GWTG-R) registry, formerly known as the National Registry of Cardiopulmonary Resuscitation, is a national, prospective, quality-improvement registry of IHCAs and is sponsored by the American Heart Association. The GWTG-R design for data collection and reliability has been described previously in detail [23]. In brief, trained research personnel at participating hospitals collect data on all IHCA patients who do not have prior do-not-resuscitate orders or cardiopulmonary resuscitation events that began outside of the hospital. Cardiac arrest is defined as pulselessness requiring chest compressions or defibrillation or both, with a hospital-wide or unit-based emergency response by acute care facility personnel. Cases are identified and data are extracted from cardiac arrest flow sheets, reviews of hospital paging system logs, routine checks of code carts, pharmacy drug records, and hospital billing charges for resuscitation medication [23].

To facilitate uniform reporting across hospitals, the registry employs Utstein-style templates for cardiac arrest, a set of standardized reporting guidelines used to define patient variables and outcomes [24,25]. Further integrity of the data is ensured through rigorous certification of data entry personnel and the use of standardized software that checks the data for completeness and accuracy [26]. All participating hospitals are required to comply with local regulatory guidelines. Because data are used primarily at the local site for quality improvement, sites are granted a waiver of informed consent under the common rule. The institutional review board at Beth Israel Deaconess Medical Center (Boston, MA, USA) reviewed the present study and determined that it did not meet the federal definition of human subject research.

### Study population

**Figure 1:**
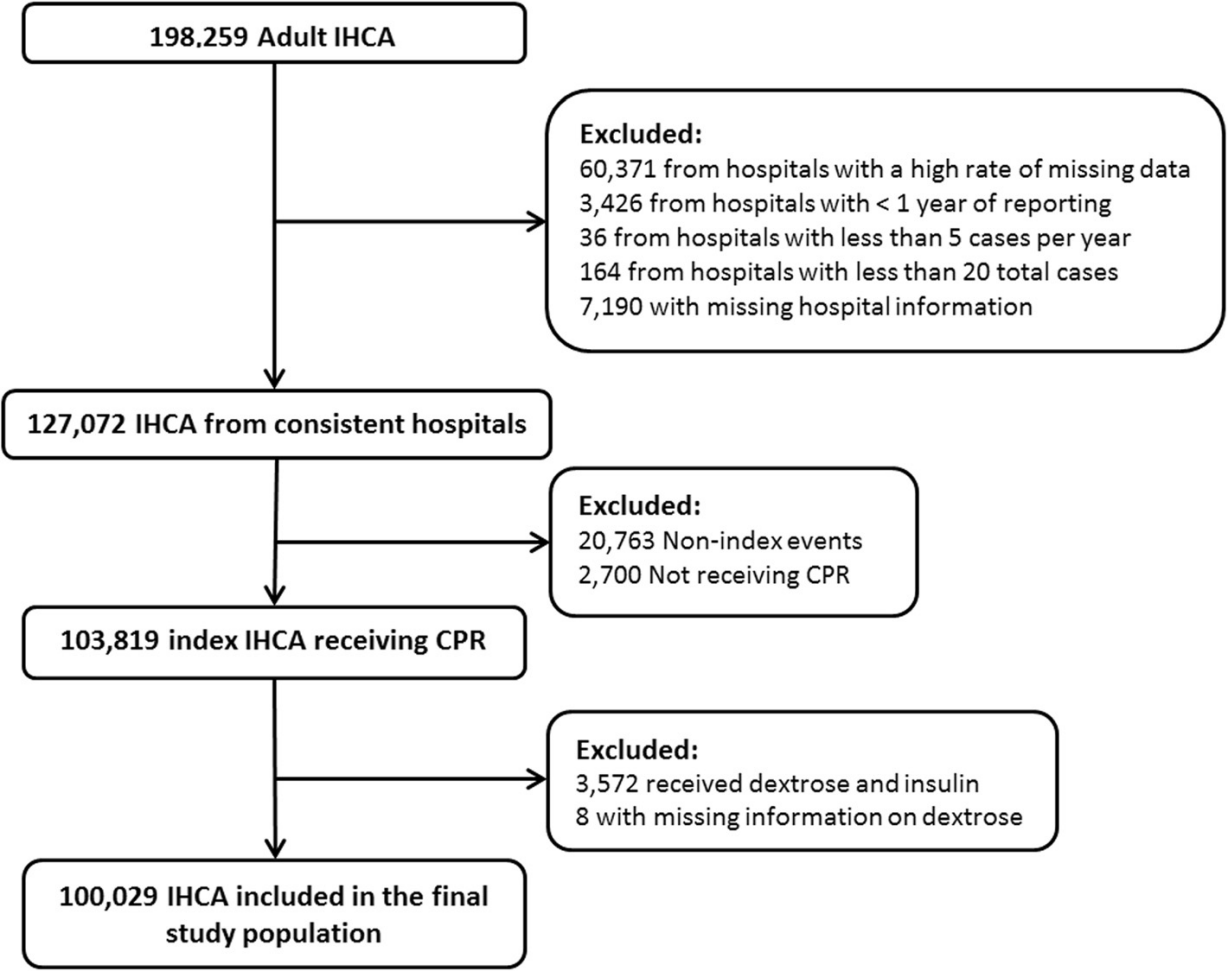
**Study population.** In total, 98,230 cardiac arrests were excluded, leaving 100,029 cardiac arrests from 349 hospitals. CPR, cardiopulmonary resuscitation; IHCA, in-hospital cardiac arrest.

Our cohort study includes data submitted to the GWTG-R registry between January 2000 and September 2010. We included all patients 18 years or older. To secure the accuracy of the data, we excluded cases from hospitals with high rates of missing data, defined as an average rate of missing data for variables in our model of more than 10%. We also excluded cases from hospitals with fewer than five cases per year, fewer than a total of 20 reported cases, less than one year of reporting, and cases with missing hospital data. Non-index events and events without initiation of cardiopulmonary resuscitation were excluded. Patients with missing data on dextrose administration and patients who simultaneously received dextrose and insulin (recommended treatment for presumed hyperkalemia) were also excluded (Figure 1).

### Study outcomes

The exposure variable, administration of dextrose, was defined as any administration of a dextrose bolus without concurrent administration of insulin during the cardiac arrest. Dextrose administration before or after the event was not included. The primary outcome of interest was survival to discharge. Secondary outcomes were good neurological outcome at the time of hospital discharge and ROSC, defined as at least 20 minutes with a palpable pulse. Neurological outcome was assessed with the use of the cerebral performance category (CPC) score, in which a CPC score of 1 indicates mild or no neurological deficit, 2 moderate cerebral disability, 3 severe cerebral disability, 4 coma or vegetative state, and 5 brain death [27]. A CPC score of 1 or 2 was considered a good neurological outcome, and a CPC score of 3 to 5 or death was considered a bad neurological outcome.

### Statistical analysis

The study population was characterized by using descriptive statistics. Categorical variables are provided in frequencies and continuous variables in means with standard deviation or medians with interquartile range (IQR), depending on the normality of the data. Differences between variables were evaluated by using chi-squared tests for categorical variables and Wilcoxon rank-sum tests for continuous variables. The change in incidence of dextrose administration over time (treated as a continuous variable for this analysis) was assessed by using unadjusted logistic regression, and the result is presented as an odds ratio (OR) with 95% confidence interval (CI).

To assess the independent association between dextrose administration during cardiac arrest resuscitation and survival to discharge, we used multivariable regression models with generalized estimating equations with an exchangeable (compound symmetry) correlation matrix to account for hospital clustering. Since the outcome was not rare (>10%), we used modified Poisson regression models with robust variance estimates to estimate risk ratios (RRs) as described by Zou [28] and Zou and Donner [29] and previously used in this cohort [2,30]. In our model, we adjusted for age, gender, race, coexisting conditions, arrest characteristics (including presumed cause of the arrest and initial rhythm), interventions during the arrest, and selected hospital characteristics (see Additional file 1: Table S1 for a full list of variables). Year of arrest was entered in the model as a categorical variable with year 2000 as the reference. All variables were chosen *a priori* on the basis of prior work and clinical reasoning [2,26,30-33]. Similar multivariate regression models were used to analyze secondary outcomes. Results from the multivariable regression models are reported as RRs with 95% CIs.

The rate of missing data in the study cohort was low (<1%) except for race (7.0%), initial rhythm (5.7%), downtime (4.7%), time of day (1.3%), and neurological outcome (2.6%). To account for missing data, we imputed the median value for patients of the same gender for all observations with missing covariates. We then did the multivariate logistic regression including the imputed values for those missing covariates. The point estimates for the variables included in the models with and without imputation were similar, and thus we have reported non-imputed models.

To determine the association between dextrose administration and post-resuscitation survival, we conducted a subgroup analysis on all patients who achieved ROSC. We performed a sensitivity analysis in which all patients with a downtime (see Additional file 1: Table S1 for a precise definition) of 5 minutes or less (to avoid confounding by potential survival bias) or with more than 10 minutes of downtime were excluded (to avoid the possibility that administration of glucose was purely a function of longer downtime). To further ensure that the patients who received dextrose were not being treated for hyperkalemia, we conducted a subgroup analysis excluding all patients who received calcium chloride or calcium gluconate. To test for effect modification, we conducted a pre-planned stratified analysis based on coexisting diabetes. We performed a number of *post hoc* analyses to assess other potential subgroup differences. We assessed interaction terms in the main model between dextrose administration and the following: cardiac cause of the arrest defined as active/evolving myocardial infarction or arrhythmia (yes/no), cardiac reason for admission (yes/no), no coexisting sepsis or hepatic insufficiency (yes/no) (that is, potential reasons for hypoglycemia in a non-diabetic patient), location of the arrest (ICU versus non-ICU), and coexisting metabolic/electrolyte abnormalities within 4 hours of the arrest (inclusive of hypoglycemia), or presumed cause of the arrest as metabolic/electrolyte abnormality (yes/no).

To assess the robustness of our findings, we performed a propensity-matched adjusted analysis to test the association between glucose administration and each outcome. For the propensity-matched analysis, we used the imputed dataset and included all variables that had been included as independent variables in the primary analysis as well as hospital center. Next, we performed a 1:3 propensity score match between patients administered and not administered glucose by using an algorithm match caliper radius of 0.10 around the propensity score. We confirmed that the matched groups were balanced by ensuring that the standardized differences between groups for each covariate were less than 10. There was a small but statistically significant difference between cases and controls for three variables (Additional file 2: Table S2). With the 1:3 propensity-matched dataset, associations between glucose administration and outcomes were assessed with the Cochran-Mantel-Haenszel test to ensure comparison between matched pairs. Using these three variables, we performed an adjusted and unadjusted conditional logistic regression analysis. There was a less than 4% change in the point estimates (adjusted versus unadjusted), and the unadjusted results are presented here. Results from the propensity-matched analysis are reported as OR with 95% CIs.

Statistical analyses were conducted with SAS software version 9.3 (SAS Institute Inc., Cary, NC, USA). All hypothesis tests were two-sided, and a *P* value of less than 0.05 was considered significant.

## Results

### Characteristics of the study population

**Figure 2:**
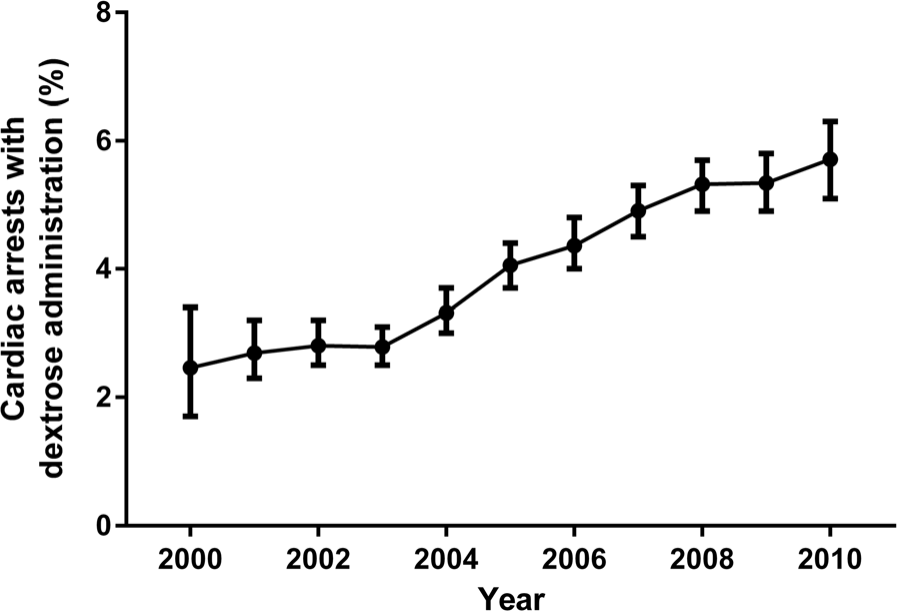
**Incidence of dextrose administration over time.** Percentage of cardiac arrests with dextrose administration over time. Error bars indicate exact binomial 95% confidence intervals (CIs). There was a steady increase in the incidence of dextrose administration from 2000 (2.5%) to 2010 (5.7%) (odds ratio 1.11, 95% CI 1.09-1.12 per year, *P* <0.001).

In total, 100,029 IHCAs from 349 hospitals were included in the main analysis (Figure 1). The median age was 69 (IQR 57–79), and 42% were female. Additional patient, arrest, and hospital characteristics are shown in Tables 1 and 2. Administration of dextrose occurred in 4,173 (4.2%) cardiac arrests. There was a significant increase in the incidence of dextrose administration from 2000 (2.5%) to 2010 (5.7%) (OR 1.11, 95% CI 1.09-1.12 per year, *P* <0.001) (Figure 2).

**Table 1:** **Characteristics of the study population according to dextrose administration**

**Characteristic**	**Received dextrose during cardiac arrest**		***P*** **value**
	**No**	**Yes**	
	**(n = 95,856)**	**(n = 4,173)**	
**Demographics**			
Age in years, median (IQR)	69 (57–79)	65 (53–77)	<0.001
Sex, number (percentage)			0.03
Female	40,306 (42.1)	1,690 (40.3)	
Male	55,550 (58.0)	2,499 (59.7)	
Race, number (percentage)			<0.001
White	68,474 (76.8)	2,527 (64.8)	
Black	17,109 (19.2)	1,208 (31.0)	
Other	3,551 (4.0)	165 (4.2)	
**Type of admission, number (percentage)**			<0.001
Medical-Non-cardiac	41,798 (43.6)	2,267 (54.3)	
Medical-Cardiac	32,031 (35.4)	1,212 (29.1)	
Surgical-Non-cardiac	10,877 (11.4)	421 (10.1)	
Surgical-Cardiac	6,188 (6.5)	185 (4.4)	
Trauma	2,777 (2.9)	73 (1.8)	
Other	272 (0.3)	14 (0.3)	
**Pre-existing conditions, number (percentage)**			
Cardiac			
Arrhythmia	31,414 (32.9)	1,188 (28.5)	<0.001
History of MI	15,864 (16.6)	584 (14.0)	<0.001
MI this admission	17,148 (17.9)	472 (11.3)	<0.001
History of heart failure	20,090 (21.0)	911 (21.9)	0.19
Heart failure this admission	17,091 (17.9)	702 (16.8)	0.08
Non-cardiac			
Respiratory insufficiency	40,001 (41.9)	1,672 (40.2)	0.03
Diabetes mellitus	28,759 (30.1)	1,717 (41.2)	<0.001
Renal insufficiency	30,784 (32.2)	1,838 (44.0)	<0.001
Metastatic/Hematologic malignancy	11,801 (12.4)	471 (11.3)	0.05
Hypotension/Hypoperfusion	26,263 (27.5)	1,107 (26.6)	0.21
Pneumonia	13,015 (13.6)	592 (14.2)	0.30
Baseline depression in CNS function	12,273 (12.8)	564 (13.5)	0.18
Metabolic/Electrolyte abnormality	15,477 (16.2)	974 (23.3)	<0.001
Septicemia	14,586 (15.3)	872 (20.9)	<0.001
Acute CNS non-stroke event	6,991 (7.3)	314 (7.5)	0.58
Hepatic insufficiency	6,724 (7.0)	431 (10.4)	<0.001
Acute stroke	3,686 (3.9)	144 (3.5)	0.18
Major trauma	3,587 (3.8)	104 (2.5)	<0.001

CNS, central nervous system; IQR, interquartile range; MI, myocardial infarction.

**Table 2:** **Arrest and hospital characteristics according to dextrose administration**

**Characteristic**	**Received dextrose during cardiac arrest**		***P*** **value**
	**No**	**Yes**	
	**(n = 95,856)**	**(n = 4,173)**	
**Location and time of the arrest, number (percentage)**			
Location			<0.001
Floor without telemetry	15,855 (16.6)	1,025 (24.6)	
Floor with telemetry	16,710 (17.4)	730 (17.5)	
Intensive care unit	45,011 (47.0)	1,597 (38.3)	
Emergency department	10,363 (10.8)	544 (13.0)	
Other	6,974 (7.6)	276 (6.6)	
Time of day			<0.001
Day (7 a.m. -10:59 p.m.)	63,897 (67.6)	2,666 (64.5)	
Night (11 p.m.-6:59 a.m.)	30,673 (32.4)	1,467 (35.5)	
Time of week, number (percentage)			0.30
Weekday (Monday-Friday)	65,269 (68.8)	2,818 (68.0)	
Weekend (Saturday-Sunday)	29,642 (31.2)	1,326 (32.0)	
Hospital-wide response called, number (percentage)	76,720 (80.0)	3,361 (80.5)	0.40
**Characteristic of the arrest**			
Monitoring, number (percentage)	78,166 (81.6)	3,053 (73.2)	<0.001
Witnessed, number (percentage)	78,262 (81.7)	3,050 (73.1)	<0.001
First rhythm shockable (VT or VF), number (percentage)	18,340 (20.3)	517 (13.0)	<0.001
Mechanical ventilation in place, number (percentage)	27,928 (29.1)	1,103 (26.4)	<0.001
Airway inserted during event, number (percentage)	51,028 (53.3)	2,680 (64.2)	<0.001
Presumed cause(s) of arrest, number (percentage)			
Arrhythmia	56,147 (58.9)	2,215 (53.4)	<0.001
Hypotension/Hypoperfusion	37,571 (39.4)	1,596 (38.5)	0.22
Active/Evolving MI	8,919 (9.4)	270 (6.5)	<0.001
Acute respiratory insufficiency	37,064 (38.9)	1,670 (40.3)	0.08
Metabolic/Electrolyte abnormality	10,809 (11.4)	860 (20.7)	<0.001
Other	7,619 (8.0)	345 (8.3)	0.46
Unknown	10,340 (10.9)	578 (13.9)	<0.001
Downtime in minutes, median (IQR)	12 (6–21)	18 (10–27)	<0.001
Medications given during the event, number (percentage)			
Amiodarone	14,806 (15.5)	703 (16.9)	0.01
Epinephrine	84,336 (88.0)	4,019 (96.3)	<0.001
Atropine	67,947 (70.9)	3,490 (83.6)	<0.001
Magnesium sulfate	7,156 (7.5)	567 (13.6)	<0.001
Lidocaine	10,152 (10.6)	381 (9.1)	0.003
Sodium bicarbonate	43,775 (45.7)	3,111 (74.6)	<0.001
Fluid bolus	28,011 (29.2)	1,452 (34.7)	<0.001
Calcium chloride/gluconate	20,188 (21.1)	1,955 (46.9)	<0.001
Norepinephrine	12,500 (13.0)	675 (16.2)	<0.001
Dopamine	23,078 (24.1)	1,037 (24.1)	0.25
**Hospital characteristics, number (percentage)**			
Bed size			<0.001
1-249	23,450 (24.2)	908 (21.7)	
250-499	44,001 (45.9)	1,836 (44.0)	
500+	28,408 (29.6)	1,429 (34.1)	
Teaching status			<0.001
Major	25,246 (26.3)	1,527 (36.6)	
Minor	33,930 (35.3)	1,162 (27.7)	
Non-teaching	36,680 (38.2)	1,484 (35.4)	
Ownership			<0.001
Private	12,247 (12.8)	540 (12.9)	
Government	14,407 (15.0)	899 (21.5)	
Non-profit	69,202 (72.2)	2,734 (65.5)	
Location			<0.001
Rural	6,274 (6.5)	216 (5.2)	
Urban	89,582 (93.5)	3,957 (94.8)	
Geographical location			<0.001
North-East	10,484 (10.9)	491 (11.7)	
South-East	26,019 (27.1)	1,125 (26.9)	
Mid-West	23,539 (21.0)	1,099 (26.9)	
South-West	19,998 (20.9)	864 (21.4)	
West	15,816 (16.5)	564 (13.5)	

IQR, interquartile range; PEA, pulseless electrical activity; VF, ventricular fibrillation; VT, ventricular tachycardia.

### Primary outcome

**Figure 3:**
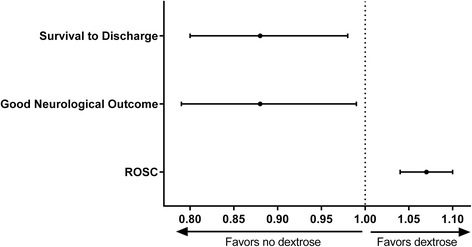
**The association between dextrose administration and outcome.** The association between administration of dextrose and survival to discharge, neurological outcome, and return of spontaneous circulation (ROSC). Adjusted risk ratios with 95% confidence intervals are shown.

Eighteen point six percent of patients survived to hospital discharge. Patients who received dextrose during cardiac arrest resuscitation had a lower rate of survival to discharge compared with patients who did not receive dextrose (RR 0.49, 95% CI 0.44-0.54, *P* <0.001). After multivariable adjustment, dextrose was still associated with a significantly decreased chance of survival to discharge (RR 0.88, 95% CI 0.80-0.98, *P* = 0.02) (Figure 3). See Additional file 3: Table S3 for the full model.

### Secondary outcomes

Fifty-eight point two percent of patients achieved ROSC, and 13.7% of patients with full data had a good neurological outcome at hospital discharge (an additional 2.6% survived but had missing data on neurological outcome). In unadjusted analyses, administration of dextrose was associated with decreased chance of ROSC (RR 0.92, 95% CI 0.88-0.96, *P* <0.001) and decreased chance of good neurological outcome (RR 0.43, 95% CI 0.38-0.49, *P* <0.001). After multivariable adjustment, dextrose administration was associated with an increased chance of ROSC (RR 1.07, 95% CI 1.04-1.10, *P* <0.001) and a decreased chance of good neurological outcome (RR 0.88, 95% CI 0.79-0.99, *P* = 0.03, Figure 3).

To further characterize the association between administration of dextrose and post-resuscitation survival, we conducted a subgroup analysis including only patients who obtained ROSC. In this subgroup, our multivariable analysis showed that administration of dextrose was still associated with both decreased chance of survival to discharge (RR 0.84, 95% CI 0.77-0.92, *P* <0.001) and decreased chance of good neurological outcome (RR 0.84, 95% CI 0.76-0.93, *P* = 0.001). In our sensitivity analysis of patients with a downtime between 5 and 10 minutes, we found that dextrose was associated with an increased chance of ROSC (RR 1.06, 95% CI 1.01-1.10, *P* = 0.008), a strong trend toward decreased chance of survival (RR 0.83, 95% CI 0.68-1.01, *P* = 0.06), and decreased chance of good neurological outcome (RR 0.78, 95% CI 0.62-0.99, *P* = 0.04).

**Figure 4:**
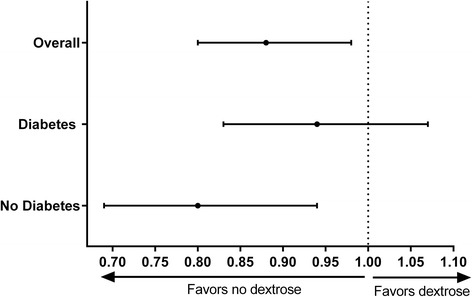
**Dextrose, diabetes status, and survival.** The association between administration of dextrose and survival to discharge was stratified by diabetes status. Adjusted risk ratios with 95% confidence intervals are shown.

We conducted a pre-planned analysis in order to investigate potential effect modification by coexisting diabetes (type I or type II). Thirty point six percent of the overall population had documented diabetes, and dextrose was more commonly administered in patients with diabetes than in patients without diabetes (5.6% versus 3.5%, *P* <0.001). There was a significant interaction between dextrose administration and diabetes status with survival as outcome (*P* = 0.02) but not with ROSC (*P* = 0.46) or good neurological outcome (*P* = 0.23) as the outcome measure. In patients with diabetes, administration of dextrose was not associated with survival to discharge (RR 0.94, 95% CI 0.83-1.07, *P* = 0.32), whereas in patients without diabetes, the administration of dextrose was associated with a decreased chance of survival (RR 0.80, 95% CI 0.69-0.94, *P* = 0.005) (Figure 4). After exclusion of patients who received calcium chloride or calcium gluconate, the association between dextrose administration and mortality remained (RR 0.88, 95% CI 0.78-0.98, *P* = 0.02). None of the other interaction terms tested (see Methods section) was statistically significant, indicating no subgroup differences.

### Results from the propensity-matched analysis

The propensity matching resulted in a successful match of 4,171 patients administered dextrose to 12,498 patients who did not receive dextrose. The groups were well matched on covariates (Additional file 2: Table S2). With our propensity-matched dataset, administration of dextrose was associated with a significantly decreased chance of survival to discharge (OR 0.80, 95% CI 0.71-0.90, *P* <0.001). Dextrose administration was likewise associated with a decreased chance of good neurological outcome (OR 0.79, 95% CI 0.68-0.91, *P* = 0.001). However, the association between dextrose administration and ROSC was not significant when the propensity-matched analysis was used (OR 1.06, 95% CI 0.99-1.13, *P* = 0.12).

## Discussion

In this large cohort study using the GWTG-R national database, we examined the association between dextrose administration and outcomes after cardiac arrest. We found that the use of dextrose during resuscitation is independently associated with lower rates of survival and unfavorable neurological outcomes. These associations remained significant even after multivariable adjustments and sensitivity analyses. The association between dextrose administration and lower rates of survival and unfavorable neurological outcomes furthermore remained in our propensity-matched analyses. In our primary analysis, dextrose administration was associated with slightly higher rates of ROSC; however, this association was no longer significant with propensity matching, making this finding difficult to interpret.

Although we cannot conclude from retrospective data why clinicians were giving dextrose during cardiac arrest, we would hypothesize that concern for hypoglycemia as the cause of arrest was likely a primary reason. Dextrose may also be administered out of concern that hypoglycemia is associated with higher mortality and potentially brain injury [34,35]. In dealing with a high-mortality event like cardiac arrest, clinicians are motivated to find and treat any potential reversible cause. With this in mind, in a truly hypoglycemic patient, the use of dextrose is probably recommended. However, studies have shown that hypoglycemia can be easily misdiagnosed in patients experiencing ischemic injuries. In current medical practice, a sample of capillary blood taken with a fingerstick blood glucose test is the quickest method to assess the possibility of hypoglycemia. However, studies have shown that fingerstick blood glucose measurements are inaccurate in patients in shock [36,37] or cardiac arrest [38]. However, the use of venous blood with a bedside glucometer is accurate and could be used if deemed necessary.

The administration of dextrose in normoglycemic or hyperglycemic patients can lead to higher blood glucose levels. Patients with hyperglycemia after cardiac arrest [14-18] and other ischemic injuries (stroke [39] and head injury [40,41]) have longer recovery times and worse neurological outcomes. Traditionally, this elevation in blood glucose has been assumed to be part of a systemic stress response. The potential mechanisms behind elevated blood glucose levels and the association with poor outcome are not well understood. Prior studies have suggested that having higher blood glucose levels during periods of ischemia increases anaerobic metabolism, promotes the conversion of pyruvate into lactate, causes intracellular acidosis, and may decrease cerebral blood flow, exacerbating cerebral ischemic injury [42-44]. Whether hyperglycemia plays a causal role in this process or is simply a marker of a systemic stress response or of dysfunctional metabolism at the cellular level is not clear.

One randomized study examined the relationship between dextrose administration after out-of-hospital cardiac arrest and neurological outcome and found no difference between the patients receiving 5% dextrose and those receiving half normal saline [45]. However, the average dose of dextrose used in this study was low (7 g) compared with the usual bolus dose (50 mL) of 50% dextrose, which contains 25 g of dextrose. To date, no study has explored the relationship between the use of a dextrose bolus during cardiac arrest resuscitation and outcome.

Our subgroup analysis revealed that although the use of dextrose was associated with higher mortality in non-diabetic patients, the association was not significant in patients with diabetes. The resilience of patients with diabetes to the deleterious effects of acute hyperglycemia has been documented [17,46]. Having chronically elevated levels of blood glucose can cause structural and functional modifications, such as a greater buffering capacity and lower cerebral pH levels after induced cardiac arrest [47]. Chronic hyperglycemia has also been found to be partially protective against cerebral hypoxia caused by acute hyperglycemia [42]. The underlying mechanism of the potential protective effects of diabetes on acute hyperglycemia needs to be clarified in future studies. Another potential explanation for our findings is the higher rate of true hypoglycemia in diabetic patients for whom administration of dextrose would likely be beneficial.

The results of our study should be interpreted in the context of the following limitations. Even though the GWTG-R registry has a rigorous training and certification process and employs standardized definitions, the data acquired by the registry may contain data integrity and validity issues. Although the GWTG-R registry is large, participation is still voluntary and this raises the potential for selection bias. Also, GWTG-R is a quality-improvement registry and was not specifically tailored to study the effects of dextrose in cardiac resuscitation. Given the observational nature of the present study, we cannot exclude the possibility that unmeasured or residual confounding remains. We were unable to identify the reason why dextrose was administered, the dosage that was given, and the timing in which it was administered. In addition, information on whether patients were receiving dextrose-containing fluid before or after the event was not available, and glucose levels were not available for any patients. It is also possible that dextrose was more likely to be administered in patients in whom initial resuscitation attempts failed, giving them a poorer prognosis. However, we believe that we addressed this potential issue in multiple ways: downtime was included in our multivariable model, and our sensitivity analyses of cardiac arrests with downtime of between 5 and 10 minutes gave largely similar results as our primary analyses. Also, potential confounding by intra-arrest variables such as prolonged downtime or non-adherence to recommended protocols cannot explain the increased chance of ROSC seen in our primary analysis. The post-cardiac arrest population is a heterogeneous group. Despite our pre-defined and *post hoc* subgroup analyses, there still could be certain subgroups of patients for whom dextrose administration is beneficial. As always, patient care should be individually tailored to the clinical situation. Finally, given the exploratory nature of our analysis, the results should be verified in a prospective manner or in a dataset with more granular data (that is, reason for dextrose administration, timing of dextrose administration, and intra- and post-cardiac arrest glucose levels) or both before any conclusions regarding clinical practice can be made.

## Conclusions

Although a causal relationship cannot be determined, our analysis shows that cardiac arrest patients receiving dextrose during resuscitation have a decreased chance of survival to hospital discharge and a decreased chance of good neurological outcome. This association seems to be driven primarily by an effect in the non-diabetic population.

## Key messages

- The association between dextrose administration during cardiac arrest and survival is unknown.
- Dextrose is used in approximately 4.2% of all in-hospital cardiac arrests, with an increasing rate from 2000 to 2010.
- Patients who received dextrose during resuscitation had lower rates of survival compared with patients who did not receive dextrose, although whether this relationship is causal remains unproven.
- This association was maintained when using multivariable regression, sensitivity analyses, and a propensity-matched analysis.
- The association between dextrose administration and poor survival seems to be driven primarily by an effect in the non-diabetic population.

## Additional files

Additional file 1: Table S1. Definitions of covariates and outcomes. Additional file 2: Table S2. Characteristics of propensity-matched groups. Additional file 3: Table S3. Primary multivariable model: association between multiple variables and survival to discharge.

## Abbreviations

ACLS: advanced cardiac life support
CPC: cerebral performance category
GWTG-R: Get With The Guidelines®-Resuscitation
IHCA: in-hospital cardiac arrest
IQR: interquartile range
OR: odds ratio
ROSC: return of spontaneous circulation
RR: risk ratio
