# Supplementary material for: The administration of dextrose during in-hospital cardiac arrest is associated with increased mortality and neurologic morbidity
Source: Crit Care. 2015 Apr 10;19(1):160. doi: 10.1186/s13054-015-0867-z (PMC4415309; doi:10.1186/s13054-015-0867-z)
Supplement: Additional file 1: Table S1. — Definitions of covariates and outcomes. [file 13054_2015_867_MOESM1_ESM.docx]

| **Demographics** | |
| --- | --- |
| **Age** | Age of the patient in years at the time of the event |
| **Sex** | Female or male (self-reported) |
| **Race** | White (*reference*), black or other (including American Indian/Alaska Native, Native Hawaiian/Pacific Islander and Asian). (Self-reported) |
| **Co-existing conditions** | |
| **Illness Category** | The most appropriate of the following illness categories at the time of the event:   - Medical-Non-cardiac – Patient with a primary diagnosis of medical illness at the time of the event that is not cardiovascular. (*reference*) - Medical-Cardiac– Patient with a primary diagnosis of medical illness that is cardiovascular at the time of the event. - Surgical-Cardiac – Patient who is post-operative following cardiac surgery at the time of the event. - Surgical-Non-cardiac– Patient who is pre-operative or post-operative with a surgical illness as the primary diagnosis that is not cardiac surgery at the time of the event. - Trauma– Patient with single or multiple traumas as the primary diagnosis at the time of the event. - Other (visitor/employer) – Neither in-patient nor outpatient, but a visitor or employee at the time of the event. |
| **Arrhythmia** | Rhythm disturbance (excluding sinus tachycardia, but including paroxysmal arrhythmias) at any time during this admission prior to the event. |
| **History of Myocardial Infarction** | Documented diagnosis of myocardial ischemia (acute coronary syndrome)/infarction prior to this admission. |
| **Myocardial Infarction this admission** | Documented diagnosis of myocardial ischemia (acute coronary syndrome)/infarction this admission. |
| **History of heart failure** | Documented diagnosis of congestive heart failure prior to this admission |
| **Heart failure this admission** | Documented diagnosis of congestive heart failure this admission |
| **Respiratory insufficiency** | Evidence of acute or chronic respiratory insufficiency within 4 hours up to the time of the event, defined by any of the following:   - PaO2/FiO2 ratio < 300 (in the absence of pre-existing documented cyanotic heart disease). - PaO2 < 60 mm Hg (in the absence of pre-existing documented cyanotic heart disease). - SaO2 < 90 %, (in the absence of pre-existing documented cyanotic heart disease); - PaCO2, EtCO2 or TcCO2 > 50 mm Hg. - Spontaneous respiratory rate > 40/min or < 5/min. - Requiring non-invasive ventilation (e.g., Bag-Valve-Mask, Mask CPAP/BiPAP, Nasal CPAP/BiPAP, negative pressure ventilation). - Requiring ventilation via invasive airway (e.g., T-piece, assist control, IMV, pressure support, high frequency). |
| **Diabetes mellitus** | Documented diagnosis of Type I or Type II diabetes mellitus |
| **Renal insufficiency** | Evidence of renal insufficiency prior to the event, defined by any of the following:   - Requiring ongoing dialysis or extracorporeal filtration therapies. - Creatinine > 2 mg/dL within 24 hours up to the time of the event |
| **Metastatic/hematologic malignancy** | Any solid tissue malignancy with evidence of metastasis, or any blood borne malignancy |
| **Hypotension/hypoperfusion** | Evidence of hypotension within 4 hours up to the time of the event, defined by any of the following:   - Systolic blood pressure < 90 or mean arterial pressure < 60 mmHg. - Vasopressor/inotropic requirement after volume expansion (except for dopamine ≤3 mcg/kg/min). - Intra-aortic balloon pump |
| **Pneumonia** | Documented diagnosis of active pneumonia, where antibiotics have not yet been started or the pneumonia is still being treated with antibiotics. |
| **Baseline depression in CNS function** | Evidence of a motor, cognitive, or functional baseline deficit (at time of system entry). |
| **Metabolic/electrolyte abnormality** | Evidence of metabolic/electrolyte abnormality within 4 hours up to the time of the event, defined by any of the following:   - Sodium < 125 or > 150 mEq/L - Potassium < 2.5 or > 6 mEq/L - pH < 7.3 or > 7.5, arterial - Lactate > 2.5 mmol/L, - Blood glucose < 60 mg/dL |
| **Septicemia** | Bloodstream infection where antibiotics have not yet been started or the infection is still being treated with antibiotics. |
| **Acute CNS non-stroke event** | Evidence of decreased mental status, delirium, or coma not due to acute stroke within 4 hours up to time of the event. |
| **Hepatic insufficiency** | Evidence of hepatic insufficiency within 24 hours up to the time of the event, defined by any of the following:   - Total bilirubin > 2 mg/dL and AST > 2x normal - Cirrhosis |
| **Acute stroke** | Documented diagnosis of an intracranial/intraventricular hemorrhage or thrombosis (during current admission). |
| **Major trauma** | Evidence of multi-system injury or single system injury associated with shock or altered mental status (during this hospitalization). |
| **Location and Time of the Arrest** | |
| **Location** | Location of the arrest. One of the following:   - Intensive Care Unit including  post-anesthesia care unit (*reference*) - Floor without telemetry - Floor with telemetry/Step-down Unit - Emergency Department - Other including ambulatory/outpatient area, cardiac catheterization laboratory, delivery suite, diagnostic/intervention area, operating room, rehab, skilled nursing or mental health unit/facility, and same-day surgical area |
| **Time of Day** | Time of arrest: day (7:00am -10:59 pm) vs. night (11:00pm – 6:59am) |
| **Time of Week** | Time of arrest: Weekday (Monday 7am – Friday 11pm) vs. weekend (Friday 11pm - Monday 7am) |
| **Year of Arrest** | Year the arrest occurred (treated as a categorical variable with 2000 as the reference) |
| **Hospital wide response called** | Hospital-wide resuscitation response activated |
| **Characteristic of the Arrest** | |
| **Monitoring status** | Patients monitored with electrocardiography, apnea monitor, bradycardia monitor and/or pulse oximetry at time of arrest |
| **Witnessed** | The onset of the cardiopulmonary arrest was directly observed by someone (family, lay bystander, employee or health care professional) |
| **First Rhythm** | First documented pulseless rhythm:   - Shockable (Pulseless Ventricular Tachycardia or Ventricular Fibrillation) - Non-shockable (Asystole or Pulseless Electrical Activity) |
| **Mechanical Ventilation in Place** | Any assisted or mechanical ventilation including CPAP/BiPAP |
| **Presumed Immediate Cause of Arrest** | One or more of the following:   - Arrhythmia - Hypotension/hypoperfusion - Active/evolving myocardial infarction - Acute Respiratory insufficiency - Metabolic/electrolyte abnormality - Other (including inadequate or obstruction of invasive/natural airway, acute pulmonary edema, and acute pulmonary embolism ) - Unknown |
| **Downtime** | Time from pulselessness to sustained return of spontaneous circulation for patients who had return of spontaneous circulation and time from pulselessness to end of event for patients who did not survive the event |
| **Insertion of airway** | Any endotracheal tube(ET) or tracheostomy tube inserted/re-inserted during the event |
| **Medications given during the event** | One or more of the following:  Amiodarone, Epinephrine, Magnesium Sulfate, Atropine, Lidocaine, Sodium Bicarbonate, Fluid Bolus, Calcium Chloride/Gluconate, Norepinephrine and/or Dopamine |
| **Hospital Characteristics** | |
| **Bed size** | Bed size of the hospital:   - 1 – 249 (*reference*) - 250 – 499 - 500+ |
| **Teaching Status** | Teaching status of the hospital   - Major (With fellowship program) (*reference*) - Minor (With residency program) - Non-teaching (No residency program) |
| **Ownership** | Ownership status of the hospital:   - Private (*reference*) - Government (including VA/Military centers) - Non-Profit (Including Church owned) |
| **Location** | Location of the hospital   - Rural - Urban |
| **Geographical Location** | Geographical Location of the hospital:   - North-East (*reference*) - South-East - Mid-West - South-West - West |
| **Outcome Variables** | |
| **Return of Spontaneous Circulation** | Documented return of adequate circulation (in the absence of ongoing chest compressions) with return of pulse/heart rate by palpation, auscultation, Doppler, arterial blood pressure waveform, or documented blood pressure lasting at least 20 minutes with no further need for chest compressions. |
| **Survival to Discharge** | Patient alive at discharge from current admission |
| **Neurological Outcome at Discharge** | Good neurological outcome:   - CPC 1: Good cerebral performance – Conscious, alert, able to work, might have mild neurologic or psychological deficit. - CPC 2: Moderate cerebral disability – conscious, sufficient cerebral function for independent activities of daily life. Able to work in sheltered environment.   Bad neurological outcome:   - CPC 3: Severe cerebral disability– Conscious, dependent on others for daily support because of impaired brain function. Ranges from ambulatory state to severe dementia or paralysis. - CPC 4: Coma or vegetative state– Any degree of coma without the presence of all brain death criteria. Unawareness, even if appears awake (vegetative state) without interaction with environment; may have spontaneous eye opening and sleep/awake cycles. Cerebral unresponsiveness. - CPC 5: Brain death– Apnea, areflexia, EEG silence, etc. |
