# Supplementary material for: The administration of dextrose during in-hospital cardiac arrest is associated with increased mortality and neurologic morbidity
Source: Crit Care. 2015 Apr 10;19(1):160. doi: 10.1186/s13054-015-0867-z (PMC4415309; doi:10.1186/s13054-015-0867-z)
Supplement: Additional file 2: Table S2. — Characteristics of propensity-matched groups. [file 13054_2015_867_MOESM2_ESM.docx]

| **Characteristic*** | **Received Glucose**  **(n = 4,171)** | **Matched Controls**  **(n = 12,498)** | **P value** |
| --- | --- | --- | --- |
| **Demographics** |  |  |  |
| Age – median years (IQR) | 66 (53 – 77) | 66 (53 – 77) | 0.60 |
| Sex – no. (%) |  |  | 0.73 |
| Female | 1,682 (40.3) | 5,002 (40.0) |  |
| Male | 2,489 (59.7) | 7,496 (60.0) |  |
| Race – no. (%) |  |  | 0.73 |
| White | 2,800 (67.1) | 8,460 (67.7) |  |
| Black | 1,206 (28.9) | 3,534 (28.3) |  |
| Other | 165 (4.0) | 504 (4.0) |  |
| **Type of Admission – no. (%)** |  |  | 0.75 |
| Medical – Non-Cardiac | 2,266 (54.3) | 6,718 (53.8) |  |
| Medical – Cardiac | 1,212 (29.1) | 3,704 (29.6) |  |
| Surgical – Non-Cardiac | 421 (10.1) | 1,201 (9.6) |  |
| Surgical – Cardiac | 185 (4.4) | 604 (4.8) |  |
| Trauma | 73 (1.8) | 233 (1.9) |  |
| Other | 14 (0.3) | 38 (0.3) |  |
| **Pre-existing conditions– no. (%)** |  |  |  |
| Cardiac |  |  |  |
| Arrhythmia | 1,188 (28.5) | 3,629 (29.0) | 0.49 |
| History of MI | 584 (14.0) | 1,808 (14.5) | 0.46 |
| MI this admission | 472 (11.3) | 1,464 (11.7) | 0.49 |
| History of heart failure | 909 (21.8) | 2,742 (21.9) | 0.84 |
| Heart failure this admission | 702 (16.8) | 2,087 (16.7) | 0.84 |
| Non-Cardiac |  |  |  |
| Respiratory insufficiency | 1,672 (40.1) | 5,094 (40.8) | 0.44 |
| Diabetes mellitus | 1,716 (41.1) | 5,097 (40.8) | 0.68 |
| Renal insufficiency | 1,836 (44.0) | 5,426 (43.4) | 0.50 |
| Metastatic/hematologic malignancy | 471 (11.3) | 1,408 (11.3) | 0.96 |
| Hypotension/hypoperfusion | 1,107 (26.5) | 3,315 (26.5) | 0.98 |
| Pneumonia | 592 (14.2) | 1,827 (14.6) | 0.50 |
| Baseline depression in CNS function | 564 (13.5) | 1,656 (13.3) | 0.65 |
| Metabolic/electrolyte abnormality | 973 (23.3) | 2,867 (22.9) | 0.61 |
| Septicemia | 872 (20.9) | 2,605 (20.8) | 0.93 |
| Acute CNS non-stroke event | 314 (7.5) | 959 (7.7) | 0.76 |
| Hepatic insufficiency | 431 (10.3) | 1,312 (10.5) | 0.76 |
| Acute stroke | 144 (3.5) | 458 (3.7) | 0.52 |
| Major trauma | 104 (2.5) | 323 (2.6) | 0.75 |
| **Location and Time of the Arrest** – no. (%) |  |  |  |
| Location |  |  | 0.65 |
| Floor Without Telemetry | 1,023 (24.5) | 2,953 (23.6) |  |
| Floor With Telemetry | 730 (17.5) | 2,233 (17.9) |  |
| Intensive Care Unit | 1,598 (38.3) | 4,912 (39.3) |  |
| Emergency Department | 544 (13.0) | 1,604 (12.8) |  |
| Other | 276 (6.6) | 796 (6.4) |  |
| Time of Day |  |  | 0.84 |
| Day (7:00am -10:59 pm) | 2,706 (64.9) | 8,087 (64.7) |  |
| Night (11:00pm – 6:59am) | 1,465 (35.1) | 4,411 (35.3) |  |
| Time of Week – no. (%) |  |  | 0.82 |
| Weekday (Monday 7am – Friday 11pm) | 2,845 (68.2) | 8,501 (68.0) |  |
| Weekend (Friday 11pm - Monday 7am) | 1,326 (31.8) | 3,997 (32.0) |  |
| Hospital wide response called – no. (%) | 3,359 (80.5) | 10,042 (80.4) | 0.80 |
| **Characteristic of the Arrest** |  |  |  |
| Monitoring – no. (%) | 3,053 (73.2) | 9,253 (74.0) | 0.29 |
| Witnessed– no. (%) | 3,050 (73.1) | 9,236 (73.9) | 0.32 |
| First Rhythm Shockable (VT or VF) – no. (%) | 517 (12.4) | 1,538 (12.3) | 0.88 |
| Mechanical Ventilation in Place – no. (%) | 1,103 (26.4) | 3,378 (27.0) | 0.46 |
| Airway inserted during event – no. (%) | 2,678 (64.2) | 8,015 (64.1) | 0.93 |
| Presumed Cause(s) of Arrest - no. (%) |  |  |  |
| Arrhythmia | 2,241 (53.7) | 6,798 (54.4) | 0.46 |
| Hypotension/hypoperfusion | 1,596 (38.3) | 4,836 (38.7) | 0.62 |
| Active/Evolving MI | 270 (6.5) | 835 (6.7) | 0.64 |
| Acute Respiratory Insufficiency | 1,670 (40.0) | 4,998 (40.0) | 0.96 |
| Metabolic/Electrolyte Abnormality | 859 (20.6) | 2,505 (20.0) | 0.44 |
| Other | 345 (8.3) | 1,033 (8.3) | 0.99 |
| Unknown | 577 (13.8) | 1,677 (13.4) | 0.50 |
| Downtime - median minutes (IQR) | 17 (11 – 26) | 16 (10 – 25) | 0.002 |
| Medications given during the event - no. (%) |  |  |  |
| Amiodarone | 703 (16.9) | 2,076 (16.6) | 0.71 |
| Epinephrine | 4,017 (96.3) | 12,131 (97.1) | 0.02 |
| Atropine | 3,488 (83.6) | 10,566 (84.5) | 0.16 |
| Magnesium Sulfate | 566 (13.6) | 1,679 (13.4) | 0.82 |
| Lidocaine | 381 (9.1) | 1,091 (8.7) | 0.42 |
| Sodium Bicarbonate | 3,109 (74.5) | 9,517 (76.2) | 0.04 |
| Fluid Bolus | 1,447 (34.7) | 4,304 (34.4) | 0.76 |
| Calcium Chloride/Gluconate | 1,953 (46.8) | 5,828 (46.6) | 0.83 |
| Norepinephrine | 675 (16.2) | 2,036 (16.3) | 0.87 |
| Dopamine | 1,037 (24.9) | 3,171 (25.4) | 0.51 |
| **Hospital Characteristics - no. (%)** |  |  |  |
| Bed size |  |  | 0.77 |
| 1 – 249 | 908 (21.8) | 2,787 (22.3) |  |
| 250 – 499 | 1,834 (44.0) | 5,447 (43.6) |  |
| 500+ | 1,429 (34.3) | 4,264 (34.1) |  |
| Teaching Status |  |  | 0.93 |
| Major | 1,526 (36.6) | 4,538 (36.3) |  |
| Minor | 1,162 (27.9) | 3,513 (28.1) |  |
| Non-teaching | 1,483 (35.6) | 4,447 (35.6) |  |
| Ownership |  |  | 0.97 |
| Private | 539 (12.9) | 1,618 (13.0) |  |
| Government | 898 (21.5) | 2,669 (21.4) |  |
| Non-Profit | 2,734 (65.6) | 8,211 (65.7) |  |
| Location |  |  | 0.76 |
| Rural | 215 (5.2) | 629 (5.0) |  |
| Urban | 3,956 (94.9) | 11,869 (95.0) |  |
| Geographical Location |  |  | 0.82 |
| North-East | 490 (11.8) | 1,512 (12.1) |  |
| South-East | 1,124 (27.0) | 3,388 (27.1) |  |
| Mid-West | 1,099 (26.4) | 3,184 (25.5) |  |
| South-West | 864 (21.4) | 2,730 (21.8) |  |
| West | 564 (13.5) | 1,684 (13.5) |  |

*IQR: Inter Quartile Range, MI: Myocardial Infarction, CNS: Central Nervous System, VF: Ventricular Fibrillation, VT: Ventricular Tachycardia, PEA: Pulseless Electrical Activity
