# Supplementary material for: The administration of dextrose during in-hospital cardiac arrest is associated with increased mortality and neurologic morbidity
Source: Crit Care. 2015 Apr 10;19(1):160. doi: 10.1186/s13054-015-0867-z (PMC4415309; doi:10.1186/s13054-015-0867-z)
Supplement: Additional file 3: Table S3. — Primary multivariable model: association between multiple variables and survival to discharge. [file 13054_2015_867_MOESM3_ESM.docx]

| **Variable*** | **Risk Ratio** | **95% CI** | **P value** |
| --- | --- | --- | --- |
|  |  |  |  |
|  |  |  |  |
| Sex (female vs. male) | 1.02 | 0.99 - 1.04 | 0.21 |
| Age (per year) | 0.99 | 0.99 – 0.99 | < 0.001 |
| Race |  |  |  |
| Black vs. white | 0.94 | 0.90 - 0.99 | 0.01 |
| Other vs. white | 1.00 | 0.92 - 1.08 | 0.91 |
| Downtime (per minute) | 0.95 | 0.95 – 0.96 | < 0.001 |
| Hospital wide response called | 0.96 | 0.92 - 1.00 | 0.06 |
| Initial rhythm (shockable vs. non-shockable) | 1.31 | 1.26 - 1.36 | < 0.001 |
| Time of day (7:00am -10:59 pm vs. 11:00pm – 6:59am) | 1.12 | 1.09 - 1.16 | < 0.001 |
| Mechanical ventilation in place | 0.67 | 0.64 - 0.71 | < 0.001 |
| Time of week (weekend vs. weekday) | 0.94 | 0.91 - 0.97 | < 0.001 |
| Co-existing conditions |  |  |  |
| Heart failure this admission | 1.00 | 0.97 - 1.04 | 0.73 |
| History of heart failure | 0.97 | 0.94 - 1.01 | 0.11 |
| Myocardial infarction this admission | 1.01 | 0.97 -1.06 | 0.57 |
| History of myocardial infarction | 1.00 | 0.97 - 1.04 | 0.86 |
| Arrhythmia | 1.10 | 1.07 - 1.13 | < 0.001 |
| Hypotension/hypoperfusion | 0.79 | 0.76 - 0.82 | < 0.001 |
| Respiratory insufficiency | 1.01 | 0.98 - 1.04 | 0.52 |
| Renal insufficiency | 0.91 | 0.88 - 0.94 | < 0.001 |
| Hepatic insufficiency | 0.69 | 0.63 - 0.74 | < 0.001 |
| Metabolic/electrolyte abnormality | 0.99 | 0.94 - 1.04 | 0.65 |
| Diabetes mellitus | 1.13 | 1.10 - 1.17 | < 0.001 |
| Baseline depression in CNS function | 0.93 | 0.88 - 0.98 | 0.003 |
| Acute stroke | 0.88 | 0.81 - 0.95 | < 0.001 |
| Acute CNS non-stroke event | 0.91 | 0.85 -0.97 | 0.002 |
| Pneumonia | 0.99 | 0.94 - 1.03 | 0.57 |
| Septicemia | 0.79 | 0.75 - 0.83 | < 0.001 |
| Major trauma | 0.89 | 0.79 - 1.00 | 0.06 |
| Metastatic/hematologic malignancy | 0.64 | 0.61 - 0.68 | < 0.001 |
| Presumed cause of the arrest |  |  |  |
| Arrhythmia | 0.96 | 0.92 - 1.00 | 0.05 |
| Hypotension/hypoperfusion | 0.83 | 0.80 - 0.87 | < 0.001 |
| Active/evolving myocardial infarction | 0.87 | 0.83 - 0.92 | < 0.001 |
| Metabolic/electrolyte abnormality | 0.97 | 0.91 - 1.03 | 0.35 |
| Acute respiratory insufficiency | 1.05 | 1.01 - 1.09 | 0.01 |
| Unknown | 0.77 | 0.71 - 0.83 | < 0.001 |
| Other | 1.13 | 1.05 - 1.22 | < 0.001 |
| Medications given/interventions during the event |  |  |  |
| Dextrose bolus | 0.88 | 0.80 – 0.98 | 0.02 |
| Amiodarone | 1.14 | 1.10 - 1.18 | < 0.001 |
| Atropine | 0.77 | 0.74 - 0.79 | < 0.001 |
| Calcium chloride/gluconate | 0.84 | 0.79 - 0.89 | < 0.001 |
| Epinephrine | 0.65 | 0.63 - 0.67 | < 0.001 |
| Dopamine | 0.85 | 0.81 - 0.89 | < 0.001 |
| Sodium bicarbonate | 0.64 | 0.61 - 0.67 | < 0.001 |
| Fluid bolus | 0.97 | 0.94 - 1.01 | 0.20 |
| Lidocaine | 1.20 | 1.16 - 1.25 | < 0.001 |
| Magnesium Sulfate | 1.12 | 1.07 - 1.18 | < 0.001 |
| Norepinephrine | 0.75 | 0.70 - 0.81 | < 0.001 |
| Insertion of airway | 1.14 | 1.11 - 1.18 | < 0.001 |
| Illness category |  |  |  |
| Medical cardiac vs. medical non-cardiac | 1.17 | 1.12 - 1.22 | < 0.001 |
| Surgical cardiac vs. medical non-cardiac | 1.79 | 1.68 - 1.91 | < 0.001 |
| Surgical non-cardiac vs. medical non-cardiac | 1.39 | 1.32 - 1.46 | < 0.001 |
| Trauma vs. medical non-cardiac | 0.98 | 0.84 - 1.13 | 0.78 |
| Other vs. medical non-cardiac | 1.47 | 1.25 - 1.72 | < 0.001 |
| Location of arrest |  |  |  |
| Emergency Department vs. ICU | 1.19 | 1.13 -1.25 | < 0.001 |
| Floor with telemetry vs. ICU | 1.15 | 1.11 - 1.20 | < 0.001 |
| Floor without telemetry vs. ICU | 0.98 | 0.92 - 1.04 | 0.44 |
| Other vs. ICU | 1.44 | 1.37 - 1.51 | < 0.001 |
| Year of arrest |  |  |  |
| 2001 vs. 2000 | 1.12 | 0.97 - 1.29 | 0.11 |
| 2002 vs. 2000 | 1.20 | 1.04 - 1.39 | 0.01 |
| 2003 vs. 2000 | 1.31 | 1.14 - 1.52 | < 0.001 |
| 2004 vs. 2000 | 1.37 | 1.18 - 1.58 | < 0.001 |
| 2005 vs. 2000 | 1.38 | 1.19 - 1.59 | < 0.001 |
| 2006 vs. 2000 | 1.47 | 1.27 - 1.71 | < 0.001 |
| 2007 vs. 2000 | 1.54 | 1.33 - 1.80 | < 0.001 |
| 2008 vs. 2000 | 1.60 | 1.37 - 1.86 | < 0.001 |
| 2009 vs. 2000 | 1.66 | 1.43 - 1.93 | < 0.001 |
| 2010 vs. 2000 | 1.79 | 1.53 - 2.10 | < 0.001 |
| Ownership |  |  |  |
| Government vs. private | 1.01 | 0.93 - 1.10 | 0.81 |
| Non-profit vs. private | 1.05 | 0.98 - 1.12 | 0.16 |
| Geographical location |  |  |  |
| South-East vs. North-East | 0.95 | 0.88 - 1.04 | 0.28 |
| Mid-West vs. North-East | 1.04 | 0.96 - 1.12 | 0.34 |
| South-West vs. North-East | 0.93 | 0.85 - 1.01 | 0.09 |
| West vs. North-East | 1.01 | 0.94 - 1.10 | 0.73 |
| Bed size |  |  |  |
| 250 – 499 vs. 1 - 249 | 1.05 | 0.99 - 1.12 | 0.10 |
| 500+ vs. 1 - 249 | 1.05 | 0.98 - 1.13 | 0.19 |
| Teaching Status |  |  |  |
| Minor teaching vs. major teaching | 0.85 | 0.80 – 0.91 | < 0.001 |
| Non-teaching vs. major teaching | 0.88 | 0.82 – 0.94 | < 0.001 |
| Location (urban vs. rural) | 0.97 | 0.90 - 1.05 | 0.49 |

*CI: Confidence interval, CNS: central nervous system, ICU: Intensive care unit
